# Quantitative Analysis of Tozadenant Using Liquid Chromatography-Mass Spectrometric Method in Rat Plasma and Its Human Pharmacokinetics Prediction Using Physiologically Based Pharmacokinetic Modeling

**DOI:** 10.3390/molecules24071295

**Published:** 2019-04-02

**Authors:** Byeong ill Lee, Min-Ho Park, Seok-Ho Shin, Jin-Ju Byeon, Yuri Park, Nahye Kim, Jangmi Choi, Young G. Shin

**Affiliations:** College of Pharmacy and Institute of Drug Research and Development, Chungnam National University, Daejeon 34134, Korea; byungill.lee.cnu@gmail.com (B.i.L.); minho.park.cnu@gmail.com (M.-H.P.); seokho.shin.cnu@gmail.com (S.-H.S.); jinju.byeon.cnu@gmail.com (J.-J.B.); yuri.park.cnu@gmail.com (Y.P.); nahye.kim.cnu@gmail.com (N.K.); jangmi.choi.cnu@gmail.com (J.C.)

**Keywords:** qualification, tozadenant, A2a receptor antagonist, PBPK modeling

## Abstract

Tozadenant is one of the selective adenosine A2a receptor antagonists with a potential to be a new Parkinson’s disease (PD) therapeutic drug. In this study, a liquid chromatography-mass spectrometry based bioanalytical method was qualified and applied for the quantitative analysis of tozadenant in rat plasma. A good calibration curve was observed in the range from 1.01 to 2200 ng/mL for tozadenant using a quadratic regression. In vitro and preclinical in vivo pharmacokinetic (PK) properties of tozadenant were studied through the developed bioanalytical methods, and human PK profiles were predicted using physiologically based pharmacokinetic (PBPK) modeling based on these values. The PBPK model was initially optimized using in vitro and in vivo PK data obtained by intravenous administration at a dose of 1 mg/kg in rats. Other in vivo PK data in rats were used to validate the PBPK model. The human PK of tozadenant after oral administration at a dose of 240 mg was simulated by using an optimized and validated PBPK model. The predicted human PK parameters and profiles were similar to the observed clinical data. As a result, optimized PBPK model could reasonably predict the PK in human.

## 1. Introduction

Parkinson’s disease (PD) is a well-known progressive neurodegenerative disease that has motor symptoms such as postural instability, tremor, bradykinesia, and rigidity. The motor symptoms of PD are caused by reduced dopamine levels in the basal ganglia [1,2]. Therefore, several drugs that can influence the amount of dopamine in basal ganglia have been used to improve the motor symptoms of PD. In particular, the precursor of dopamine, l-DOPA (l-dihydroxy-phenyl-alanine), has been used as a gold standard for over 40 years [3].

However, while l-DOPA therapies are effective for the first several years, long-term treatment with l-DOPA therapies result in several adverse effects, including motor fluctuation and dyskinesia [1,3,4,5,6]. Therefore, alternative therapies are needed for the treatment of PD patients taking long-term l-DOPA therapies.

Recently, adenosine A2a receptor antagonists have been reported to be potential alternative therapies for the treatment of PD [7,8,9,10,11,12,13,14]. Adenosine A2a receptors are co-localized to striatopallidal neurons with dopamine D2 and D3 receptors that are part of the indirect basal ganglia output pathway from the striatum to the thalamus [7,15,16]. Adenosine A2a receptors activate adenylate cyclase to form cAMP, while the dopamine D2 receptors have an opposite effect. Also, adenosine A2a receptor agonists decrease the binding affinity of dopamine to the dopamine D2 receptors [17,18,19]. In other words, adenosine A2a receptor antagonists produce biological effects the same as dopamine D2 and D3 agonists by influencing the activity of the indirect basal ganglia output pathway, which plays an important role in the regulation of spontaneous motor activity [20]. Currently, a number of adenosine A2a receptor antagonists have been studied. Xanthine-based drugs such as caffeine and theophylline are non-selectively bound to the adenosine A2a receptor [21,22]. With the effort to increase the potency and selectivity to the adenosine A2a receptor, modified xanthine-based drugs such as KF17837, 8-(3-Chlorostyryl)caffeine (CSC), 3,7-demethyl-1-propargylxanthine (DMPX), and istradefylline, as well as non-xanthine-based drugs such as ZM241385, SCH58261, CGS15943, and CP66713 have been developed [2,23,24,25,26]. Many adenosine A2a receptor antagonists have shown various efficacies in preclinical studies, such as haloperidol-treated rodents, 6-hydroxydopamine (6-OHDA)-lesioned rats, 1-methyl-4-phenyl-1,2,3,6-tetrahydropyridine (MPTP)-treated primates, and clinical trials in PD [27]. One of these adenosine A2a receptor antagonists is tozadenant [3,7,8,13,28]. Tozadenant is a selective adenosine A2a receptor antagonist that can be administered orally. Tozadenant was shown to be tolerable and effective as an adjunct to levodopa in PD patients with motor fluctuations in the phase IIb clinical trial [3].

In this study, we describe the development and qualification of an LC-MS/MS method for tozadenant in rat plasma. The method was successfully applied to its pharmacokinetic (PK) studies in rats. Also, in vitro absorption, distribution, metabolism, and excretion (ADME) properties such as microsomal metabolic stability and plasma protein binding were measured. Based on these in vitro and preclinical in vivo data, the rat PK profiles and parameters of tozadenant were predicted using physiologically based pharmacokinetic (PBPK) modeling. PBPK modeling is a sophisticated mathematical model that divides the body into physiological and anatomical compartments reflecting systemic specific physiological properties and the drug’s physicochemical properties [29,30,31,32]. PBPK modeling is widely used to predict ADME/PK properties of drugs from in vitro and/or in vivo input data and simulates PK profiles and parameters of drugs in animals or humans [33,34,35]. In particular, usage of the PBPK model in the prediction for human pharmacokinetics has been recognized as a powerful tool, and its utilization has increased significantly in recent years [36,37,38,39,40,41].

Finally, we predicted the human PK profiles and parameters of tozadenant using the optimized PBPK model obtained from in vitro/preclinical data and compared with the clinically obtained PK data of tozadenant in human [13].

## 2. Results

### 2.1. Method Qualification x

**Figure 1:**
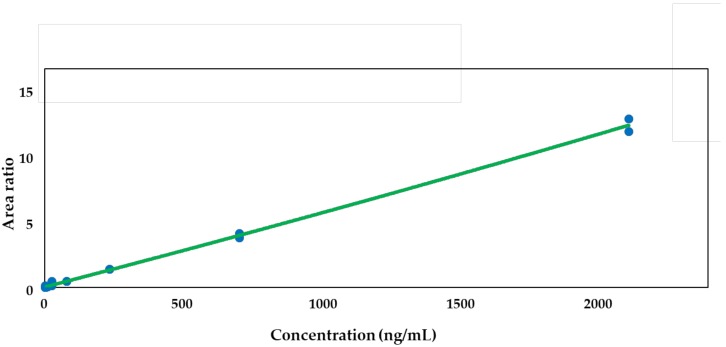
A typical calibration curve (r = 0.99524, range = 1.01 − 2200 ng/mL) for tozadenant in rat plasma. *Y* axis; area ratio = [analyte peak area/verapamil (ISTD) peak area].

Calibration curves with eight points in the range of 1.01–2200 ng/mL in duplicate were freshly prepared for all data sets. The quadratic regression using the ratios of peaks versus concentrations was weighted by 1/concentration^2^. The acceptance of the curve was conducted from the coefficient of determination (r) values for the calibration curves, and the result was ≥0.99 for tozadenant. Although a quadratic regression was used in this LC-MS study for the compensation of the matrix effect as well as the ion saturation of the electrospray ionization process, other regressions—including a linear regression—would also be applicable to the analysis of tozadenant as long as they meet the acceptance criteria of the calibration curve. In this study, a quadratic regression showed better coefficient of determination than any other regressions. Figure 1 shows the calibration curve of tozadenant.

**Figure 2:**
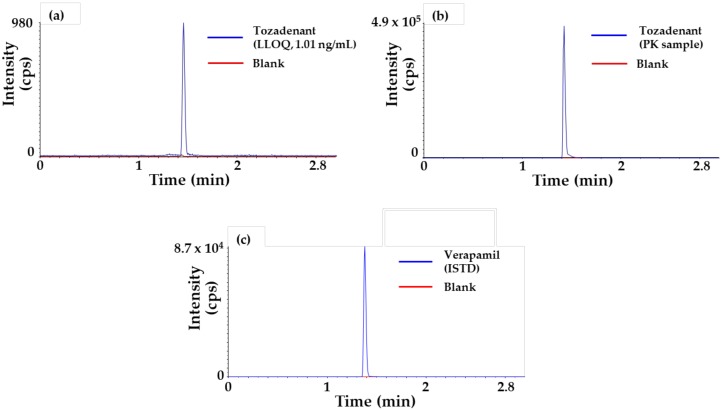
Typical chromatograms of tozadenant and verapamil (ISTD). (**a**) Lower limit of quantification (LLOQ), 1.01 ng/mL of tozadenant, (**b**) plasma sample collected at 2 min after intravenous administration of 1 mg/kg in rat of tozadenant and (**c**) verapamil with extracted blank matrix.

Representative chromatograms of tozadenant [lower limit of quantification (LLOQ), 1.01 ng/mL] and verapamil (ISTD) samples are also shown in Figure 2.

This assay provided a decent sensitivity to cover the rat PK studies throughout the PK time course up to 24 h with a dose level of ≥1 mg/kg. Although no lower concentration below the LLOQ (1.01 ng/mL) was explored due to the sufficient calibration curve range for the rat PK samples, the limit of detection (LOD) with signal-to-noise (S/N) ratio = 3 and the limit of quantification (LOQ) with S/N = 10 would be 0.05 ng/mL and 0.1 ng/mL, respectively, and this information would be useful for method development if the lower dose PK studies were conducted. The performance of the assay was checked by evaluating the intra-run and inter-run accuracy (% Acc) and precision (% CV) for each quality control (QC) level in triplicate, and the results are presented in Table 1. The qualification run met the criteria of acceptance with ±25% accuracy and precision for all QC levels.

The dilution integrity assessment was performed with dilution QC samples in triplicate, and the results are shown in Table 2. The dilution QC samples also met the criteria of acceptance with ±25% accuracy and precision. As a result, the dilution integrity assessment showed that the five times dilutions using blank rat plasma were successful.

The results of the preliminary stability assessments are shown in Table 3.

The preliminary stability QC samples also met the acceptance criteria of ±25% accuracy and precision. As a result, tozadenant in rat plasma was stable for 12 h at room temperature (RT), for 14 days at −20 °C, and through three cycles of freeze-thaw process at −20 °C with acceptable accuracy and precision values. Also, tozadenant in stock solution was stable for 28 days at −20 °C.

### 2.2. In Vitro Experiments

#### 2.2.1. Plasma Protein Binding

The plasma protein binding of tozadenant was independent to the concentration of 0.1 and 1 µg/mL for both rats and humans. Therefore, the mean unbound fraction (Fup) values from each concentration level were used in the Gastroplus™ as the input data for each species. The Fup values of tozadenant in rat and human plasma were 26.63% and 26.72%, respectively.

#### 2.2.2. Microsomal Metabolic Stability

The results of microsomal metabolic stability are shown in Table 4.

The Cl_int, in vitro_ values of tozadenant were similar in the range of 0.5–2 mg/mL concentration of liver microsomes. Therefore, the mean Cl_int, in vitro_ values were used, and the results were 0.0021 and 0.0008 mL/min/mg microsomal protein in two species of liver microsomes, respectively. The scaled Cl_int_ values of tozadenant were 3.78 and 0.99 mL/min/kg, and the extrapolated hepatic clearance values were 3.53 and 0.95 mL/min/kg in rats and humans, respectively.

### 2.3. Application for a Pharmacokinetic Study in Rats

**Figure 3:**
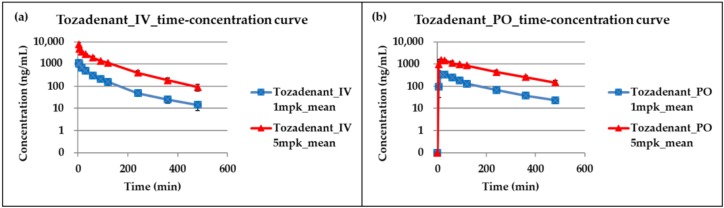
Time-concentration profiles of tozadenant after (**a**) intravenous and (**b**) oral administration at a dose range of 1 to 5 mg/kg in rats.

This LC-MS/MS method was successfully applied to a tozadenant pharmacokinetic study in rats, and Figure 3 shows the time-concentration profiles of tozadenant after intravenous and oral administration. Table 5 shows pharmacokinetic parameters calculated from this study. The results show that the increase in area under the curve (AUC) and maximum plasma concentration (C_max_) of tozadenant was dose-dependent at a single intravenous or oral administration at a dose range of 1 to 5 mg/kg, and the average oral bioavailability (F) was 69.43% in rats.

### 2.4. Prediction of Plasma Concentration-Time Profiles Using the PBPK Model

**Figure 4:**
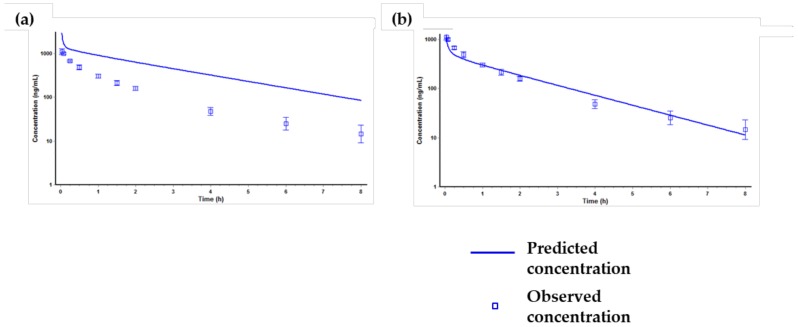
Predicted and observed time-concentration profiles of tozadenant after a single intravenous administration at a dose of 1 mg/kg in rats. (**a**) PK profile predicted only using the input parameters in Table 6, (**b**) PK profile predicted after optimizing the kidney clearance, the Kp value of the liver and the kidney.

The simulation results of the PBPK model for tozadenant after a single intravenous administration at a dose of 1 mg/kg in rats are presented in Figure 4.

**Figure 5:**
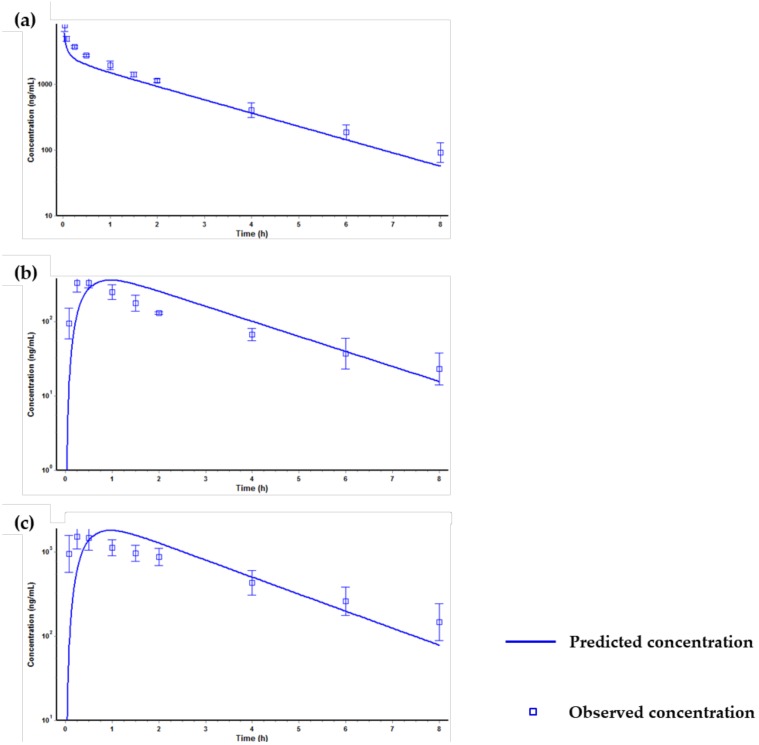
Predicted and observed time-concentration profiles of tozadenant after optimization, (**a**) intravenous administration at a dose of 5 mg/kg, (**b**) oral administration at a dose of 1 mg/kg, (**c**) oral administration at a dose of 5 mg/kg in rats.

The first simulation using only the input parameters (Table 6) showed significant difference between the predicted and the observed value (Figure 4a). Specifically, the predicted AUC_last_ value was much higher than the observed AUC_last_ value. We confirmed that the clearance (Cl) and volume of distribution (Vd) values predicted from the first simulation showed no resemblance to the observed parameters. The Cl and Vd values from the observed PK data were 14.36 mL/min/kg and 1.63 L/kg, respectively, but the values from the predicted PK data were 0.422 mL/min/kg and 0.7 L/kg, respectively. The difference in the Cl value was considered to be due to the fact that Gastroplus™ predicted the in vivo Cl value based on the Cl_int, in vitro_ used as an input parameter and thus did not match the observed in vivo Cl value. Therefore, in order to match the in vivo Cl value, the kidney clearance was optimized as extra hepatic clearance [42,43]. Also, the difference of Vd value was optimized by optimizing the Kp (distribution coefficient) value of the liver and the kidney by using the optimization module in Gastroplus™. After optimization of Cl and Vd values, the predicted PK profile and parameters were similar to the observed PK profile and parameters (Figure 4b). Then, the optimized PBPK model was validated via other in vivo PK data, and the results are shown in Figure 5.

**Figure 6:**
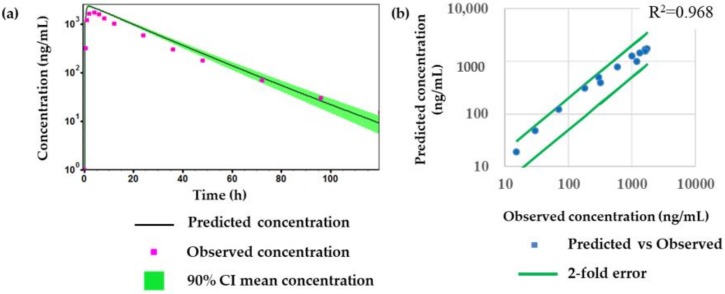
Comparison of observed values and predicted values for tozadenant; (**a**) predicted and observed time-concentration profile of tozadenant after oral administration at a dose of 240 mg in humans, (**b**) correlation between observed concentration and predicted concentration.

Figure 5 shows that the optimized PBPK model was well fitted when compared with the predicted PK profiles with the observed PK profiles at different dose levels (1 and 5 mg/kg) or by different dosing routes (intravenous or oral dosage). After the validation of the PBPK model, the human PK of tozadenant after oral administration at a dose of 240 mg was simulated using the optimized and validated PBPK model, and the results are shown in Figure 6 and Table 7.

Overall, Figure 6 and Table 7 show that the optimized and validated PBPK model reasonably matched the PK profile and parameters of tozadenant in the human clinical study.

## 3. Discussion and Conclusions

In this study, an LC-MS/MS method was newly developed and applied for the determination of tozadenant in rat plasma. The calibration curve was good enough to cover the concentration range from 1.01 to 2200 ng/mL for tozadenant using a quadratic regression. This LC-MS/MS method was sensitive and selective enough to determine tozadenant in rat plasma samples and was successfully used for various in vitro and in vivo PK studies. Using several measured in vitro and in vivo ADME properties, a PBPK model in rats was reasonably constructed using Gastroplus™ and was applied to predict the PK of tozadenant in humans. The results of the human PK prediction showed that prediction fold error value was within two folds between the predicted and the reference clinical data, which implies that the predicted human PK values are considerably acceptable from the industry’s standpoints [30,39,42].

There is a constant increase for human PK predictions using available PBPK software tools, such as Gastroplus™ (Simulations Plus, Lancaster, CA, USA), Cloe^®^ Predict (Cyprotex, Macclesfiled, UK), PK Sim^®^ (Bayer Technology Services, Leverkusen, Germany), and Simcyp^®^ simulator (Simcyp, Sheffield, UK) [29,33,39,42,44,45]. The PBPK model using these software tools is expected to be an effective method to predict the dose range and the dose escalation procedures to facilitate the clinical study design, and it is expected to be a helpful method to reduce times and costs for clinical trials associated with drug-drug interactions, food effects, etc.

## 4. Materials and Methods

### 4.1. Chemicals and Reagents

Tozadenant was purchased from MedChem Express (Monmouth Junction, NJ, USA). Verapamil, which was used for internal standard (ISTD), was purchased from Sigma-Aldrich (St Louis, MO, USA). HPLC-grade acetonitrile (ACN) was purchased from Honeywell Burdick & Jackson (Ulsan, Korea), and HPLC-grade distilled water (DW) was purchased from Samchun Chemical (Gyeonggi-do, Korea). Dimethyl sulfoxide (DMSO) and formic acid were obtained from Daejung Chemical (Gyeonggi-do, Korea). All other chemicals were commercial products of either analytical grade or reagent grade, and no further purification was used.

### 4.2. Preparation of Stock Solution, Calibration Standard, Qaulity Control, and Internal Standard

One mg/mL tozadenant in DMSO was used for the stock solution and stored in the refrigerator at −20 °C until use. After that, sub-stock solution with 0.1 mg/mL tozadenant in DMSO was made by diluting the stock solution using blank DMSO. Serial dilution of the sub-stock was done to make eight calibration standard solutions using DMSO. The final concentrations for each of the eight calibration standard samples were made in blank rat plasma to be 1.01 3.02, 9.05, 27.2, 81.5, 244, 733, and 2200 ng/mL, respectively. Three levels of QC samples were also made at 15 ng/mL (QC low), 165 ng/mL (QC medium), and 1820 ng/mL (QC high) with blank rat plasma. Verapamil was used as an ISTD. The ISTD was prepared at 1 mg/mL verapamil in DMSO and stored in the refrigerator at −20 °C until use. The final ISTD spiking solution containing 20 ng/mL of verapamil was prepared in ACN.

### 4.3. Sample Preparation

Four µL of the calibration standard (STD) or QC working solutions were added to 20 µL of blank rat plasma for STD or QC samples, while 4 µL of make-up solutions (DMSO) were added to blank rat plasma for blank samples. For study samples, 4 µL of make-up solutions (DMSO) were also added to 20 µL of rat PK study samples to assure the same matrix conditions as the STD and the QC samples. Then, 100 µL of the final ISTD spiking solution containing 20 ng/mL of verapamil was added to STD, QC, and study samples for protein precipitation. Then, the samples were mixed by vortexing for 1 min and centrifuged at 10,000 rpm for 5 min. Following the centrifugation, 50 µL of supernatant was transferred to another tube and diluted by adding 100 µL of DW. Then, the mixture was transferred to an LC vial for LC-MS analysis.

### 4.4. LC-MS/MS Conditions

The LC-MS/MS system for this experiment consisted of an Agilent 1290 Infinity 2 LC system equipped with a high speed binary pump (G7120A), a vial sampler (G7129B), a thermostatic-column (G7116B), and a Sciex QTRAP 6500^®^ mass spectrometer. The LC column used for this method was a Waters CORTECS^®^ C18+ column (2.1 × 50 mm, 2.7 µM). A linear LC gradient profile was employed using two mobile phases (aqueous mobile phase A: 0.1% formic acid in DW, organic mobile phase B: 0.1% formic acid in ACN); 0.4 mL/min was set for the flow rate, and 10 μL was injected. The LC gradient profile was set at 5% organic mobile phase B for the first 0.5 min and then increased to 95% B at 1.1 min. It held 95% B for another 0.2 min and then decreased to the initial condition in 0.1 min for column re-equilibrium. The LC-MS/MS run time per sample was 3 min. Tozadenant and ISTD were eluted at 1.45 and 1.38 min, respectively.

The QTRAP 6500^®^ mass spectrometer in the positive ion mode using a TurboIonSpray^®^ ion source was used. The instrument conditions were as follows: source temperature set at 500 °C with a curtain gas flow of 35 L/min (GS1 and GS2); ion spray voltage set at 5500 V; declustering potential 130 V for tozadenant and 93 V for verapamil; and collision energy 36 V for tozadenant and 30 V for verapamil, respectively. The quantification was performed using the following multiple reaction monitoring (MRM) transitions of the respective [M + H]^+^ ions: *m*/*z* 407.2 → 292.1 for tozadenant, 455.2 → 165.2 for verapamil, respectively.

### 4.5. Method Qualification

Method qualification was carried out with a “fit-for-purpose” approach. The qualification run contained duplicate standards with eight concentrations and QCs with three levels of concentrations. The acceptance criteria for standards and QCs in the qualification run were within ±25% precision and accuracy. A calibration curve was made by establishing the quadratic regression function with the equation y = ax^2^ + bx + c after 1/concentration^2^ weighting. In addition, two blank plasma samples were run.

The dilution integrity assessment was carried out to demonstrate the concentrations above the upper limit of quantification (ULOQ) could be analyzed for the acceptable concentration in the calibration curve after proper dilution with blank rat plasma. The dilution integrity assessment was performed with dilution QC samples in triplicate. The acceptance criteria for dilution integrity were within ±25% precision and accuracy.

Preliminary stability assessments were also conducted for four experimental conditions including short-term, long-term, stock solution, and freeze-thaw. Each of the preliminary stability assessments was performed with QC samples in triplicate. Stock solution stability was evaluated by comparing peak intensities between 28 day-old stock solution and the freshly prepared stock solution. The stability assay for the freeze-thaw cycles was evaluated by comparing the stability sample processed with three cycles of freeze-thaw at −20 °C with the freshly prepared samples. Short-term matrix stability and long-term matrix stability were determined at RT for 12 h and −20 °C for 14 days, respectively. The acceptance criteria for all discovery stage stability tests were within ±25% precision and accuracy in this study.

### 4.6. In Vitro Experiments

#### 4.6.1. Plasma Protein Binding

The plasma protein binding of tozadenant (0.1 and 1 µg/mL) was determined by equilibrium dialysis in pooled Sprague-Dawley (SD) rat and human plasma. Equilibrium dialysis was performed using the Thermo Scientific™ Rapid Equilibrium Dialysis (RED) device system with 8 kDa molecular weight cutoff (Thermo Scientific, Rockford, IL, USA). Then, 300 µL of plasma sample containing tozadenant (0.1 and 1 µg/mL) was dialyzed against 500 µL of phosphate buffered saline (PBS) for 4 h at 37 °C. After the dialysis incubation time, the plasma samples were transferred to cluster tubes, and the same volume of PBS was added. In the same way, the PBS samples were transferred to cluster tubes, and the same volume of blank rat plasma was added to make the same matrix.

Then, all samples were prepared with the protein precipitation method followed by the LC-MS/MS analysis. The Fup of tozadenant in plasma was measured by calculating the ratio of tozadenant in PBS samples to plasma samples.

#### 4.6.2. Microsomal Metabolic Stability

The in vitro microsomal metabolic stability of tozadenant was measured under the following final conditions: tozadenant (1.5 µg/mL), rat and human liver microsomes (0.5–2 mg/mL), β-Nicotinamide adenine dinucleotide hydrate (NADPH) (2 mM), Uridine 5′-diphosphoglucuronic acid (UDPGA) (5 mM). All incubations were performed in triplicate at 37 °C and initiated by adding cofactor solutions containing NADPH and UDPGA to liver microsome solutions for 3 min pre-incubation. After pre-incubation, tozadenant was added to the incubation mixture. All incubations were quenched with the final ISTD spiking solution containing 20 ng/mL of verapamil in ACN at 0, 15, 30, and 60 min after incubation. Then, all samples were prepared by the protein precipitation method and analyzed by the LC-MS/MS method.

The in vitro intrinsic clearance, Cl_int, in vitro_ (mL/min/mg), was calculated by the following equation:

Cl_int, in vitro_ = (0.693/T_1/2_) × (1/C_microsomal protein concentration_) (1)

where T_1/2_ (=0.693/k) was calculated by the slope (k) of the log-linear regression analysis of the remaining amount (%, the ratio of sample peak area/ISTD peak area) versus time profiles. The Cl_int, in vitro_ values were also calculated by scaling the in vitro data to the in vivo ones for rats and humans.

The in vivo intrinsic clearance, Cl_int_ (mL/min/kg), was obtained by following equation:

Cl_int_ = Cl_int, in vitro_ × (mg microsomal protein/g liver) × (g liver/kg body) (2)

where the values of hepatic microsomal protein concentrations were 44.8 and 48.8 mg microsomal protein/g liver in rats and humans, respectively, and the values of liver concentrations were 40 and 25.7 g liver/kg body weight in rats and humans, respectively [46].

Then, the hepatic clearance (Cl_H_) was calculated by the well-stirred model [47,48]:

Cl_H_ = (Q × Cl_int_)/(Q + Cl_int_) (3)

where the values of Q, the hepatic blood flow, were 55.2 and 20.7 mL/min/kg in rats and humans, respectively.

### 4.7. Application for a Pharmacokinetic Study in Rat

SD rats (300 ± 10 g, *n* = 4) were fasted for 12 h prior to drug administration. After dosing intravenously or orally with tozadenant at 1 and 5 mg/kg, approximately 150μL of blood samples were drawn into the heparinized tubes at 0, 2, 5, 10, 20, 40, 60, 90, 120, 240, 360, and 480 min and were immediately centrifuged at 10,000 rpm (9600 g) for 5 min. Then, the supernatant plasma samples were transferred to another eppendorf tube and stored in the deep freezer at −20 °C until analysis.

Animal experiments abided the animal care protocol (no. CNU-01104) approved from Chungnam National University. All procedures related to animal experiments also abided by the guidelines established by the Association for Assessment and Accreditation of Laboratory Animal Care International (AAALAC International).

### 4.8. Pharmacokinetic Data Analysis

Pharmacokientic parameters were calculated using non-compartmental analysis (NCA) with Phoenix WinNonLin software (version 6.5; Pharsight Corporation, Mountain View, CA, USA). The maximal plasma concentration (C_max_) and the area under the plasma concentration-versus-time curve from time zero to the last time point (AUC_0-t_) and extrapolated to infinity (AUC_0-∞_) were calculated. The absolute oral bioavailability (F) was also calculated as F = (Dose_iv_/Dose_po_) × (AUC_po_/AUC_iv_) × 100 (%).

### 4.9. Prediction of Plasma Concentration-Time Profiles Using the PBPK Model

The GastroPlus™ (version 9.5; Simulations Plus, Inc, Lancaster, CA, USA) PBPK model was used for all simulations in rats and humans. The PBPK model adopted in this study was made up of 14 compartments represented by various tissues of the body, which were linked by the venous and arterial blood circulation. The perfusion-limited tissue model was used, in which the kinetics of drug to tissue were determined by the Kp values (distribution coefficient) of each tissue. The physicochemical and ADME properties of tozadenant, such as pKa, logP, permeability, solubility, and blood/plasma ratio, were predicted by the ADMET predictor module in GastroPlus™ based on the structure of tozadenant. In addition to the predicted physicochemical and ADME properties, the experimentally observed in vitro data, such as unbound fraction and in vitro intrinsic clearance, were used as input data for the development of the PBPK model. The values of the input parameters for simulations of tozadenant are summarized in Table 1.

The PBPK model was optimized by using the in vitro data and in vivo PK data obtained by intravenous administration at a dose of 1 mg/kg in rats. The optimization module in GastroPlus™ was used for the PBPK model optimization. Then, other in vivo PK data in rats were used to validate the PBPK model. The optimized and validated PBPK model using in vitro and in vivo data from rats was scaled up to fit human physiology. Finally, the human PK of tozadenant was simulated in the oral dosage of 240 mg using the achieved PBPK model. The predicted PK results were then compared to the observed reference clinical data.

## Figures and Tables

**Table 1:** Statistics of quality control (QC) levels from the qualification run for tozadenant in rat plasma.

**Run Number**	**Statistics**	**QC Low** **(15.04 ng/mL)**	**QC Medium** **(165.46 ng/mL)**	**QC High** **(1820 ng/mL)**
1	Mean n % Acc % CV	15.94 3 106.01 6.81	171.91 3 103.90 1.32	1802.27 3 99.03 4.62
2	Mean n % Acc % CV	14.95 3 99.38 6.19	157.76 3 95.34 1.13	1684.91 3 92.58 0.24
3	Mean n % Acc % CV	15.32 3 101.88 4.34	163.48 3 98.80 1.78	1807.70 3 99.32 0.51
Inter-run	Mean n % Acc % CV	15.40 9 102.42 5.84	164.38 9 99.35 3.95	1764.96 9 96.98 4.15

% Acc = inter-run accuracy, % CV = precision.

**Table 2:** The dilution integrity assessment for tozadenant in rat plasma.

Statistics	Dilution QC (6600 ng/mL)
Mean	6268.4
n	3
% Acc	94.98
% CV	4.3

**Table 3:** The preliminary stability assessments for tozadenant in rat plasma.

Assessment	Statistics	QC Low (15.04 ng/mL)	QC Medium (165.46 ng/mL)	QC High (1820 ng/mL)
Short-term (room temperature, 12 h)	Mean n % Acc % CV	16.48 3 109.6 5.9	165.6 3 100.09 1.91	1804.08 3 99.13 2.9
Long-term (−20 °C, 14 days)	Mean n % Acc % CV	14.87 3 98.87 2.1	156.13 3 94.36 3.87	1648.15 3 90.56 0.34
Freeze-thaw (−20 °C, 3 cycles)	Mean n % Acc % CV	14.68 3 97.63 6.64	155.64 3 94.07 6.18	1733.81 3 95.26 1.47
Stock (−20 °C, 28 days)	Mean n % Acc % CV	15.65 3 104.08 4.41	175.44 3 106.03 2.68	1814.65 3 99.71 4.31

**Table 4:** The microsomal metabolic stability of tozadenant in rat and human liver microsomes.

Species	Cl_int, *in vitro*_ (mL/min/mg)	Cl_int_ (mL/min/mg)	Cl_H_ (mL/min/mg)
Rat	0.0021 ±0.0003	3.78 ±0.67	3.53 ±0.58
Human	0.0008 ±0.0002	0.99 ±0.23	0.95 ±0.20

**Table 5:** Pharmacokinetic parameters of tozadenant after intravenous (IV) and oral (PO) administration at a dose range of 1 to 5 mg/kg in rats (*n* = 4).

PK (Pharmokinetic) Study	Dose (mg/kg)	T_1/2_ (min)	T_max_ (min)	C_max_ (ng/mL)	AUC_last_ (min × ng/mL)	AUC_INF_ (min × ng/mL)	Clearancev(Cl) (mL/min/kg)	Volume of Distribution(Vd) (L/kg)
IV	1	139.26 ±40.05	2	1118.92 ±164.61	67142.34 ±6521.56	70342.35 ±7723.57	14.36 ±1.71	1.63 ±0.33
	5	99.69 ±19.07	2	7820.27 ±1627.22	431241.43 ±38843.52	445005.24 ±45762.56	11.31 ±1.13	1.28 ±0.09
PO	1	147.91 ±57.85	22.5 ±8.66	368.97 ±60.06	48958.98 ±5784.28	54665.08 ±10852.83		
	5	144.84 ±42.76	27.5 ±23.98	1666.3 ±448.52	279450.32 ±64941.07	313549.48 ±88341.03		

**Table 6:** Input parameters used in GastroPlus™ for the physiologically based pharmacokinetic (PBPK) model simulation of tozadenant.

Parameters	Values
Molecular weight (g/mol)	406.5
pKa ^1^	3.28, 4.7, 10.81
Log P ^1^	1.96
Permeability (cm^2^/s) ^1^	1.62
Solubility at pH 7 (mg/mL) ^1^	0.28
Blood/plasma concentration ratio (Rbp) ^1^ in rat and human ^1^	0.82
Unbound fraction (Fup) in rat and human (%) ^2^	26.63, 26.72
Cl_int in vitro_ in rat and human (mL/min/mg) ^2^	0.0021, 0.0008

All input parameters were calculated by GastroPlus™; ^1^ predicted values, ^2^ measured values.

**Table 7:** Predicted and observed PK parameters of tozadenant at a dose of 240 mg in humans. Prediction fold errors are also included.

PK Parameter	AUC_last_ (µg × h/mL)	C_max_ (µg/mL)	T_max_ (h)	T_1/2_ (h)
Observed	35.0	1.74	4	15
Predicted	49.6	1.8	2.8	17.4
Prediction fold error	1.4	1.0	0.7	1.2
